# Sustaining transformations: changing marine governance, environmental meaning, and ‘left behind’ Brexit narratives on the Yorkshire East Coast

**DOI:** 10.1007/s40152-022-00290-1

**Published:** 2022-12-06

**Authors:** Anna S. Antonova

**Affiliations:** Rachel Carson Center for Environment and Society, LMU Munich, Leopoldstr. 11a, D-80802 Munich, Germany

**Keywords:** Just transformations, Marine governance, Environmental narratives, Discursive institutionalism, Brexit

## Abstract

Transformations to sustainability are frequently framed as key to blue growth, but they often engender complex consequences for communities. This article illustrates the role of environmental meaning in these processes through the lens of the Brexit vote on the Yorkshire East Coast. Based on discursive institutionalist analysis of narrative materials from semi-formal interviews conducted in 2017 alongside textual documentation from media, policy, and regional archives, I trace connections between transforming marine governance regimes, environmental meaning, and the British relationship with the EU from the Cod Wars to today. The transformation towards ecosystem-based management in British maritime governance post UNCLOS III left local communities feeling ‘left behind’ not only economically but also in terms of marginalised local meanings of place, labour, and environment. The Brexit vote, in this context, shows the multivalence of transformational processes and the importance of considering environmental meaning as part of their just execution.

## Introduction

As global environmental change increasingly requires societal and political transformations, the question of how to execute the necessary governance transitions justly as well as successfully has come to the forefront of scholarly debate (Bennett et al. 2021; Feola et al. 2021; Thomas 2021). Scholars examining transformations to sustainability have shown that the success of these processes requires an equal commitment to tackling politically and socially complex factors (Feola et al. 2021; Manuel-Navarrete and Pelling 2015). They have emphasised the necessity of reflecting on historical contexts (Parsons and Nalau 2016) and types of future thinking involved in transformational processes. The question of who might be left behind by the transition process while planning environmental policy for the future has been particularly relevant for coastal communities, not only because of their heightened vulnerability to various climate change impacts (Pörtner et al. 2019) but also because the realm of marine governance has already seen one of the arguably most fundamental paradigm transformations executed globally in recent decades (Juda 2001; Spalding and de Ycaza 2020).

In this article, I take the relationship between environmental beliefs, changing marine governance, and Brexit on the British coast in order to illustrate the far-reaching consequences of failing to account for the ‘left behind’ in environmental transformations. It has been argued before that Brexit turned as much on the appeal of narratives, identities, and socio-cultural degradation as on concrete politics or economics (Bolet 2021; Gamble 2018; Spencer and Oppermann 2020). Meanwhile, in the coastal context, observers have highlighted that the fishing industry’s visibility in the debates in the lead-up to the EU Referendum in 2016 far surpassed its 0.5 per cent share of the national gross domestic product (Carpenter 2017; Jack 2020). However, the analytical interest that the fisheries industry has drawn as a result has tended to focus on the specifics of fishery governance and UK-EU trade relations (see Barnes and Rosello 2016; Billiet 2019; Phillipson and Symes 2018; Stewart and Carpenter 2021; Stewart et al. 2022). With this article, I place a much-needed focus on the role that environmental beliefs and identities and their underrepresentation in a marine governance paradigm shifting towards ecosystem management played in British coastal communities’ overwhelming tendency to vote Leave. The case I examine highlights the significance of considering environmental meaning as a vital element of any transformation to sustainability. It shows that failing to do so may have far-reaching societal and political consequences.

As this article will show, an important underlying cause for Brexit on the coast was British coastal communities’ sense of being left behind, economically but also in terms of their environmental beliefs, by decades-long fundamental transformation in marine governance. I suggest that British coastal communities’ sentiments of being poorly served by policy change originated in part from the flawed implementation of a drastically new form of environmental management, one which advanced an idea of environment divergent from the traditional sentiments of environment and society ingrained in the national and local psyche. The resulting clash of visions over environment helped sway British coastal communities’ views of Europe in the lead-up to Brexit. Since environmental norms have been adopted by the EU increasingly throughout the decades as a form of supranational legitimacy within and beyond its member states (Antonova and van Dam 2022; Burns 2019; Kelemen and Vogel 2010; Lightfoot and Burchell 2004; Manners and Murray 2016), the EU became an easy target for discontent with how environmental policies were implemented in the UK. Thus, I also demonstrate how the sustainability transformation in marine governance on the British coast engendered societal and geopolitical consequences that extended beyond the realm of environmental policy.

## Methods and approach

**Fig. 1:**
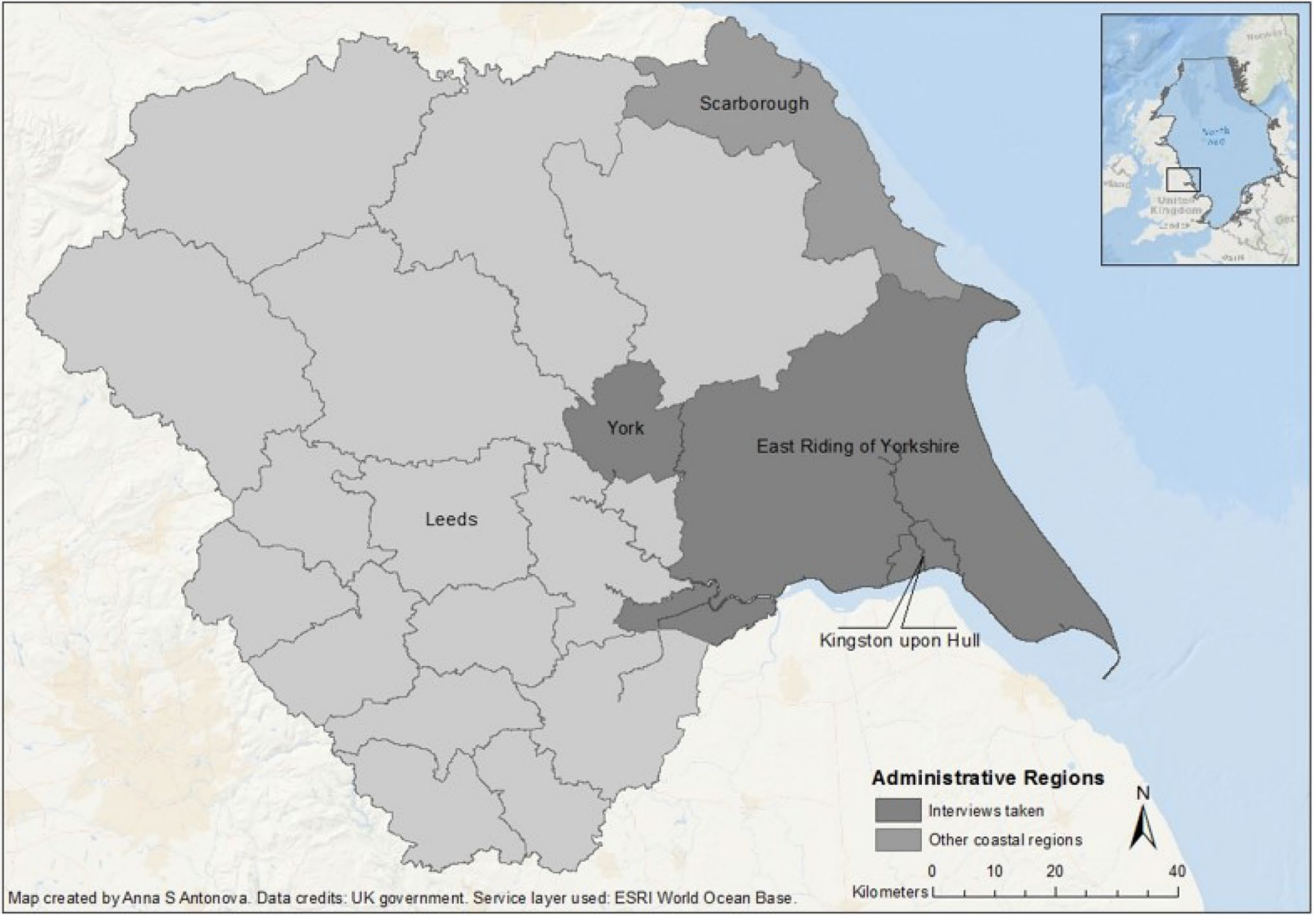
Map of Yorkshire showing its coastal administrative units (Scarborough Borough, the East Riding of Yorkshire district, and the city of Kingston upon Hull), as well as indicating where in Yorkshire interviews were taken

My observations in this article draw on my analysis of a wide range of narrative materials collected as part of a larger research project that investigated and compared societal and environmental transformations in two European coastal contexts, the Yorkshire East Coast and the Bulgarian Black Sea shore (2020). This article draws on my work in Yorkshire, Britain’s largest county, located in North England with a coastline on the North Sea that stretches from Spurn Point on the mouth of the Humber Estuary (to the south) to Staithes Beck (to the north). I conducted the majority of my fieldwork in this context in 2017 within the unitary authority of the East Riding of Yorkshire, although I also took interviews in York and Kingston upon Hull (commonly abbreviated as Hull) (see Fig. 1).

The wider project took narratives as its main unit of analysis (Yin 2003). To ensure qualitative data triangulation (as prescribed by Flick 2018) and thereby a variety of perspectives to inform my observations and deepen my insights, my methodology relied on analysis of numerous sources of narrative, including original interviews, media pieces, legal and political documents, literature, and archival materials. These materials were all chosen through a purposive snowball sampling approach (Bernard 2006; Farquharson 2005). My selection and analysis of the interview materials and of the textual narratives cross-pollinated and informed each other throughout the project (e.g. participants recommending relevant texts or textual narrative sources suggesting important perspectives to solicit in interviewing), ensuring a continuous evolution of theoretical insights throughout in accordance with grounded theory approaches (Glaser and Strauss 1967; Charmaz 2006). Nevertheless, sampling differed slightly between participants and textual sources of documents.

As part of my broader fieldwork for this project, I conducted fifteen original semi-structured open-ended interviews in Yorkshire in 2017 with representatives of local institutions, community members, policy makers, scholars, activists, and members of NGOs (see Table 1).

**Table 1:** Interviews taken in Yorkshire per sector as part of the project

Sector	Local community members	Activists/advocates/NGO members	Researchers/academics	Local institutions/policy makers	Total
Interviews	3	4	3	5	15

These interlocuters were selected through a purposive snowball sampling modelled on Farquharson’s reputational snowball method (Farquharson 2005). Starting with a couple of key informants—selected because preliminary research indicated they would be well placed in the communities of interest to the project—I solicited suggestions for other participants, asking for individuals whose voices others in the sample considered representative of different but in their view key perspectives on the societal and environmental transformations in the Yorkshire coastal context. I deliberately asked participants to suggest not only others whose views aligned with theirs but also ones whose perspective might differ from or even oppose theirs. The ‘reputational’ aspect of this purposeful sampling technique (Farquharson 2005) meant that I approached participants whose names had been suggested to me most frequently. I followed up on and approached subsequent recommendations until interlocuters began proposing individuals I had already spoken to, thus indicating a point of saturation.

The textual narratives I analysed were likewise chosen through a purposive snowball sampling approach, one that, as previously mentioned, ran alongside the selection of interview participants. Here, the selection process was somewhat more flexible in line with the continuous analysis methods favoured by grounded theory approaches (Charmaz 2006). As part of my research, I selected and analysed textual materials before, during, and after fieldwork. For the Yorkshire context, these narrative sources included archival materials obtained from the Hull History Centre, various UK and EU policies, legal documents, and reports, as well as numerous media pieces, opinions, and other textual documents.

For this article, my analysis of these varying narrative materials relies on a discursive institutionalist approach in order to explain the complicated relationship between the UK’s changing marine governance, environmental meaning in coastal communities, and the outcome of the EU Referendum in the British coastal context. As an approach, discursive institutionalism traces the political impact of ideational power, defined as ‘the capacity of actors (whether individual or collective) to influence other actors’ normative and cognitive beliefs’ (Carstensen and Schmidt 2016, 318). In this way, discursive institutionalist analysis can fruitfully combine tracing legal history developments with the examination of contemporary narratives and their impact on politics (Fischer and Gottweis 2013; Schmidt 2010, 2015). I approach this task by presenting the legal history of the UK’s fishing relations with the EU together with the changing ideas and narratives of maritime governance that have at least partially affected contemporary British coastal communities’ discontent with the EU. In short, I ground the ocean governance transformation to sustainability in the concrete context of marine policy change as part of UK-EU relations, focusing especially on the consequences for local coastal communities.

**Fig. 2:**
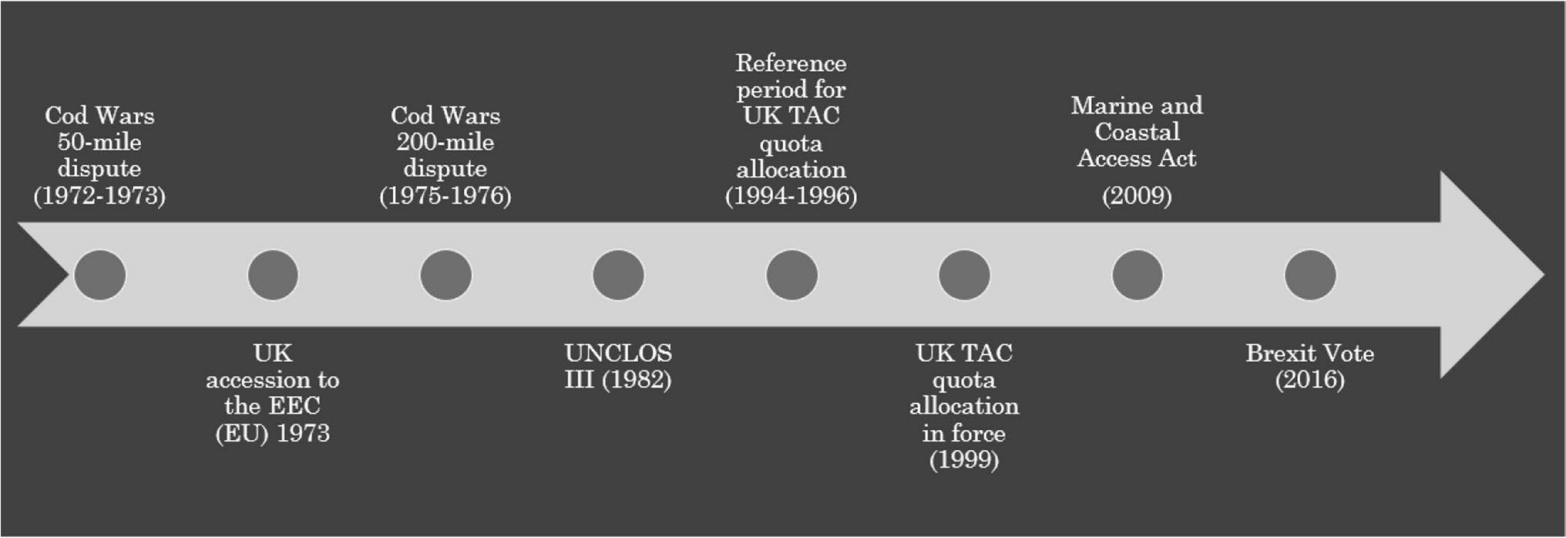
Major historical and policy events in Britain and Yorkshire’s marine governance policy transformations

More specifically, I examine Brexit as a function of ‘left behind’ sentiments in UK coastal communities against the backdrop of decades-long marine governance change. In evoking language of the ‘left behind’, my analysis follows Ford and Goodwin’s (2014a, b) postulation that social change and cultural identity have been vital for driving right wing policy in the UK. This theorisation was later evoked by Goodwin and Heath (2016) to show how cultural factors, and specifically the sense of being ‘left behind’ by generational change as well as by economics, informed the Brexit vote across Britain. I narrow in on the shifting cultural values associated with fishing and the marine environment in the UK. For coastal communities, the fishing industry has long represented a source of not only income but also of local pride and identity. Throughout the Cod Wars—a series of maritime conflicts between Iceland and the UK during the Cold War, each prompted by Iceland’s jurisdiction claims over its adjacent maritime space (Guðmundsson 2006)—the UK government supported these values of the industry as national ones, legitimising them. However, with the adoption of the Third United Nations Convention on the Law of the Sea (UNCLOS III) in 1982 and throughout the UK’s accession negotiations with the EEC/EU, other priorities came to the fore. Over the subsequent decades, UK marine governance underwent more than one paradigmatic change. As the ‘Environmental meaning and marine governance’ section will show, UNCLOS III and accession to the EEC/EU (and thus the Common Fisheries Policy, CFP) resulted in a fundamental shift in the UK’s management of fisheries to a single-species approach and the setting of total allowable catches (TACs) (see also Symes 1992). With the wider global turn towards integrated coastal management, however, the UK commenced a subsequent transformation towards ecosystem-based management, adding priorities in marine conservation and offshore wind energy (Cardwell and Thornton 2015; Fletcher et al. 2014). While the rest of this article will explore these policy shifts in more depth—focusing on the local experience in Yorkshire and the resultant attitudes towards the EU—the trajectory of events is briefly represented in Fig. 2.

As this article will go on to demonstrate, this rapidly changing marine policy landscape left coastal communities feeling underrepresented by their government. Meanwhile, the EU’s role in advancing and representing these new forms of marine management as part of its own normative formation (Antonova 2015; Manners and Murray 2016) has made it an easy ideational target for British politicians to shift discontent onto despite being the ones responsible for overlooking coastal communities. Thus, the article analyses the discursive role that fisheries and the marine environment have played in UK-EU relations from Britain’s original accession negotiations onwards. In so doing, the article illustrates how discourses and ideas of environment can become a key driver for politics and international relations beyond the specific context of sustainability transformations.

The article proceeds as follows. First, by focusing on the example of Yorkshire, I contextualise Brexit narratives, sentiments, and events on the British coast in the lead-up to the EU Referendum. I especially draw out the discursive, as well as economic, significance of fisheries in the lead-up to the EU Referendum, relating this to Ford and Goodwin’s theorisations of the ‘left behind’ (2014a, b). In the section that follows, I examine archival evidence documenting the role that environmental beliefs associated with fisheries, Britain’s Cod Wars with Iceland, UNCLOS III, and the resulting change in global marine governance all played into UK relations with the EU (then the European Economic Community, or EEC) at the time of the British accession negotiations. This evidence suggests that coastal communities felt the effects of losing fishing opportunities in terms of identity as well as economics, while various actors alternatively overlooked or exploited their sentiments for political gain. The penultimate section shows how these dynamics set the tone for coastal communities feeling underserved by changing marine governance from the 1990s onward. Pro-Leave narratives in 2016 resonated with British coastal communities who had felt increasingly crowded out by new maritime uses and flawed implementation of various marine and environmental governance policies in the previous decades. Together, these two sections show how Britain’s marine governance paradigm transformation was political, and its relative successes and failures both turned on its ability to take different environmental sentiments into account. I then sum up what these discursive patterns suggest for necessity of considering environmental meaning as part of sustainability transformations.

## Brexit and ‘left behind’ sentiments on the British coast

Environmental change and policy transformation can often have a large effect on already vulnerable communities. Across the UK, a majority of seaside towns have experienced decades of cumulative decline. Government commissioned studies from the 2000s to today have consistently established that coastal communities face an increased likelihood of experiencing social and economic isolation due to factors like physical distance, poor housing and services, transience, and the outward migration of youth, altogether resulting in higher levels of deprivation in twenty-six out of thirty-seven principal and in twenty-two out of thirty-seven smaller seaside towns across the UK (Communities and Local Government Committee 2007; Beatty et al. 2008, 2011). In 2019, a House of Lords reports observed that seaside communities and particularly smaller coastal towns in Britain tended to feel ‘overlooked and unloved by the Government, local councils, service providers and businesses alike’ and thus ultimately ‘isolated, unsupported and left behind’ (Select Committee on Regenerating Seaside Towns and Communities 2019, 6). And in 2021, a year into the COVID-19 pandemic, the annual report of the UK’s chief medical officer found that English coastal towns had some of the country’s worst health outcomes, even when adjusted for economic and demographic factors (Whitty 2021).

These dynamics can be illustrated well in parts of coastal Yorkshire. As of 2019, for example, both Bridlington and Hull contained areas listed in national statistics within the 0.1 percentile of deprivation in the UK (Ministry of Housing, Communities, and Local Government 2019). Yet the consequences extend well beyond economics. This truth was demonstrated by the results of the EU Referendum, in which Yorkshire coastal communities overwhelmingly voted to Leave, with the Leave vote taking 60.4 per cent of the voters in the East Riding of Yorkshire, 61.99 per cent of those in Scarborough, and 67.62 per cent of those in Hull (Reed 2016). Speaking from this context, local policy officer ‘Helen’^1^ reflected that the local deprivation levels had a direct impact on how the community saw itself, remarking that she thought ‘as a country we’re losing pride […] I think it’s even in themselves, I just don’t think people have any pride in anything anymore. Certainly not in the town that they live in’ (interview with author, 2017). Observing how this loss of pride impacted even everyday behaviours—she gave the example of the increased tendency to throwing away of rubbish on the beach—Helen saw the consequences of economic deprivation extending beyond income to sense of society and environment.

Against this backdrop, the significance of the fishing industry includes, and yet also goes beyond, regional economics. Reflecting on fisheries and Brexit exemplifies the divide between national and local economics that helped drive the Referendum’s outcome, since, on a regional level, fishing tends to offer a vital source of revenue for coastal communities statistically more likely to face economic decline, isolation, and deprivation. To illustrate, the shellfishing industry in Bridlington—a town featuring Britain’s 44th most deprived area in 2019 (out of 32,844)—supplied £10.5 million in landing value in 2018 alone (Ministry of Housing, Communities, and Local Government 2019; Marine Management Organization 2019). In this context, therefore, fishing represents a regionally significant source of income.

Just as importantly, however, the high value and success of the fishing industry serve locally also as a source for community pride. The shellfishing industry on the Yorkshire coast, for example, is locally lauded for being able to ‘land more lobsters than anywhere in Europe’ (‘William’, fisheries advocate, interview with author, 2017). Yet, as participants in the context remarked, knowledge of this significance generally does not penetrate beyond the local context. ‘Connor’ observed that the industry often ‘exists on the edge’, clarifying that ‘fishing communities are not generally historically well heard, well listened to […] normally you wouldn't see politicians coming and campaigning around fishing ports’ (‘Connor’, fisheries advocate, interview with author, 2017). In this sense, the political and media attention given to fisheries throughout the Brexit campaigns elevated coastal communities to a level of visibility directly in contrast to their usual political and social isolation from national attention. Connor spoke directly to this conclusion, observing that the ‘romanticised’ image of fishermen as ‘old men with big beards knitting jumpers and singing sea shanties while going out for mackerel’ or grouped together into generic national symbolism of ‘red telephone boxes and cycling vicars and cricket on the green and fishing boats’, the true value in this representation as part of the Brexit debate was in the community’s being represented at all: ‘it’s still helpful in that people are aware we’re there, that it’s an industry that exists and […] that it’s a community that exists’ (2017). For local small-scale fisherman James, this potential visibility meant, for example, that he could hope to get more quota, even if he did not believe that ‘our fleet will get back to what it was, I just don’t see it somehow’ (interview with author, 2017). And although contemporary analyses had suggested that precisely the type of fishing like James’ on which coastal communities rely—that is, small-scale, non-recipient of quota—would be the hardest hit by Brexit (Carpenter 2017), participants observations’ show that for many in these communities, the visibility that the fishing industry could attain through the debates and subsequent policy was a vital part of the point.

The importance of the fishing industry and its local value being recognised and seen on the national level speaks directly to illustrating Goodwin and Heath’s thesis that Brexit had tapped into an entanglement between economic deprivation and cultural isolation in small communities (Goodwin and Heath 2016). Their analysis draws on Ford and Goodwin’s earlier work in *Revolt on the Right* and particularly on its insight into the ways that generational, long-term change in the social and cultural *milieu* contribute to sentiments of feeling ‘left behind’ (Ford and Goodwin 2014a). Similar sentiments extend beyond Britain, having been found in follow-up work in other European countries (Hobolt 2016). However, materials I collected in Yorkshire suggest that for coastal communities, specifically, these findings apply directly not only because of economic or general socio-cultural dynamics but also because fishing, along with the values it evokes for its communities, has been as much subject to shifting societal and political meanings and the subsequent associations of these with the EU.

## Environmental meaning and marine governance: the political symbolism of fisheries in the UK through the Cod Wars and EEC negotiations

The local significance of the fishing industry described in the previous section is further complicated by the multifaceted semantic role that fisheries have played in contemporary UK national identity politics. As this section will show, fishing as a symbol has been strategically utilised by a succession of British political figures from the earliest stages of the UK’s negotiations for accession into the EEC. Simultaneously, however, fishing has also stood at the heart of an internal tension about the UK’s identity as a legal actor in the contemporary international stage, including in its relationship with Europe. To some extent, this tension both parallels and underlies the common inheritance of imperial thinking identified by historian Robert Saunders in his analysis of the British relationship with Europe (Saunders 2020). On the one hand, a vision of British leadership grounded in its history of oceanic and imperial power had long benefited from the freedom of the seas principle, which supported the extension of geopolitical power by large naval states like itself (Steinberg 2001). As part of that vision, long-distance trawlers fishing in distant waters were seen by the UK government and by coastal communities as a source of pride through their pioneering spirit, as well as because of their service in the Second World War (Jóhannesson 2004). On the other hand, in the postcolonial context, voices within the British political elite have increasingly seen the UK’s global role as best propagated through multilateral action (Saunders 2020). British leadership, in this sense, has been framed not in terms of its independence but instead in terms of its propagation of normative goals on the global stage, that is leading by example and through cooperation (Saunders 2020). In this context, Britain has striven to be perceived as a leader in normative environmental governance, not only in terms of its own policies on climate, clean energy, or marine management, but also through its active influence on the development of EU environmental policies and directives (Burns et al. 2016).

This internal tension became particularly evident during the Cod Wars—a series of conflicts in which the UK, on behalf of its long-distance fishing fleets, contested Iceland’s increasing claims to adjacent waters from four to twelve (1958–1961 dispute), fifty (1972–1973 dispute), and eventually two-hundred (1975–1976 dispute) nautical miles (Guðmundsson 2006). The Cod Wars hold an important place in the modern history of international ocean law because they helped influence the outcome of UNCLOS III in terms codifying the 200-mile exclusive economic zone (EEZ) as a universal legal principle (Guðmundsson 2006). They also show the UK’s evolving relationship to international ocean law at the time. Throughout the disputes, the UK took seemingly contradictory positions in different global flora. In supporting its long-distance fleets, the government argued that any resolution of its conflict with Iceland must be ‘consistent with [the British fleet’s] traditional interest and acquired rights in and current dependency on those fisheries’ (United Kingdom v. Iceland 1974, 8) and asserted that Iceland had ‘no authority […] in international law, whether conventional or customary’ for its ‘unjustifiable and invalid’ extended claims (United Kingdom v. Iceland 1974, 10). In confidence, as archival sources show, voices from within the government considered the disputes to have escalated ‘far beyond any rational or reasonable level of confrontation for this amount of fish’, highlighting that ‘NATO is threatened; Iceland appears ready to break diplomatic relations with Britain and many lives have been put in real jeopardy’ (Conservative Research Department 1976, 1). And the same internal voices demonstrated their awareness that the British government’s position on the conflict was ‘inconsistent with British pronouncements and actions in other fora’—namely, the ongoing negotiations for the third UNCLOS, through which the UK intended to lay the same claim to a 200-mile EEZ that it was objecting to in the case of Iceland (Conservative Research Department 1976, 4–5).

As first the Cod Wars and then the establishment of EEZs through the negotiations for UNCLOS III progressively closed off Icelandic waters to British long-distance trawlers, the question of alternative fishing opportunities would be increasingly raised as part of the UK’s negotiations for accession with the EEC. Speaking in 1971, in the context of the 12-mile dispute and the first signs of the upcoming 50-mile dispute, Yorkshire representative MP Patrick Wall claimed that ‘agree[ing] terms for the fishing industry’ would ‘considerably influence my vote’ with respect to the EEC accession (Wall 1971, 1). Moreover, British agreement to ‘the need for a [Brussels-based] fishing policy at all’ was made contingent on renegotiating the founding six member states’ original (1970) Common Fisheries Policy (CFP); concurrently, voices from within the UK government argued that the future new version of the CFP ‘must also reflect […] the loss of distant waters by our fleets and […] protect those communities which rely so heavily on fishing’ (Conservative Research Department 1977, 1–3). Thus, British scepticism about the CFP—which would, decades later, be healthily fed and debated during the Brexit debates—actually began with concern over the policy’s ability to provide fishing opportunities to the UK’s long-distance trawling communities.

Despite concerns that ‘the future is extremely bleak for our distant water fleet which is the largest within the Community’ (Conservative Research Department 1977, 2) throughout the accession negotiations, however, fishing communities’ interests were just as often used by British politicians as fodder for internal political fighting over different interpretations of Britain’s role and legal position in the context of changing international ocean law. For instance, political lines were drawn with respect to how the conflict with Iceland may influence British relations with the EEC. In 1975 Roy Hattersley, Minister of State for Foreign and Commonwealth Affairs for the Labour Government, saw potential danger in allowing EEC mediation in the conflicts: ‘We are prepared to use and enjoy the good offices of any friendly Power or organisation which can persuade the Icelandic Government to negotiate with us [but] we must make it clear that some of these matters are proper for us, not for the European Economic Community […We do] not want the E.E.C. to extend its competence to an area which we believe is proper for Great Britain and for this government’ (Hattersley 1975, n.p.). Contemporaneous analysis briefs from the Conservative Research Department, conversely, saw in EEC mediation the opportunity not only to ‘bring the dispute to an early end’ but also ‘enhance Britain’s international image and strengthen her hand in the vital renegotiation of the EEC’s Common Fisheries Policy’ and recommended that ‘The Government should be criticised for its reluctance to accept mediation’ (Conservative Research Department 1976, 4). Similarly, although Conservative and Labour governments had largely pursued similar policies throughout the Cod War disputes, the same confidential brief recommended to Conservative party members that they publicly press the Labour government for its handling of the Cod Wars dispute and suggested that ‘The Government should be pressed to explain the preparations it is making to secure the interests of the fishing industry under the expected new legal regime. The explanation would probably be inadequate and leave the Government open to criticism’ (Conservative Research Department 1976, 5).

These exchanges and communications demonstrate not only British politicians’ growing awareness that the legal regime was changing but also the conflicting views on what role the UK should take within the new regime and how that role should shape its relations with the EEC. They also show, however, that the actual communities most concretely affected by these changes in the UK were at least in part left in the background of these deliberations. By 1980, for instance, the long-distance trawling community in Hull—one of the key communities involved in the Cod Wars conflicts—would define its situation as ‘desperate’ and reprimand the government for shifting the blame onto the EEC: ‘the port takes the view it is simply not good enough for the Minister to shelter behind the excuses that such negotiations [for more fishing opportunities] must be conducted by the EEC’ (Hull Fishing Industry 1980). Ultimately, as I heard from participants off the record in 2017, fishing communities came to feel that they had been pawned off by their government during the original membership negotiations with the EEC. The idea that the UK deliberately sacrificed its fishing interests in the 1970s in the name of a more favourable overarching accession arrangement has been documented in scholarship (Phillipson and Symes 2018, p. 169) and finds resonance with similar sentiments in the wake of the Trade and Cooperation Act (‘UK Fisheries Sold out in Brexit Deal, Industry Body Says’ 2021). It was an idea, moreover, that lent itself to being exploited in British politics as easily in the wake of the Cod Wars and the EEC accession negotiations as it would be later in debates and campaigns leading up to Brexit.

## Changing marine governance, the normative clout of the EU, and the ‘left behind’

The profound transformational in environmental governance in terms of the legal management of the oceans through UNCLOS III played a significant role in both politics pertaining to British coastal communities and in their perceptions of the EEC/EU. The EEC’s exclusive supranational competence in fisheries emerged in response to the UNCLOS III paradigm shift and drew its justification from it (Antonova 2015, 2016). This resulted in an early and strong ideational association between the EU and the newly established legal framework for oceanic governance. This normative link became stronger still in the 1990s as narratives of ‘green Europe’ and their perceived benefits to EU integration helped motivate the EU’s strengthened competences over environmental policy (Kelemen and Vogel 2010; Manners and Murray 2016). Meanwhile, a series of EU policies and directives—from the CFP through the Birds (Directive 2009/147/EC) and Habitats (Council Directive 92/43/EEC) Directives to the Water Framework Directive (Directive 2000/60/EC) and the Marine Strategy Framework Directive (Directive 2008/56/EC)—resulted in explicit policy change through their domestic iteration in the UK (Fletcher et al. 2014). With the introduction of the Marine and Coastal Access Act in 2009, the UK government simultaneously responded to EU directives and to the increasing demands of an increasing number of marine activities developing in the UK’s waters (Fletcher et al. 2014). Thus, the EU became strongly implicated in what Seamus, a local policy officer, called ‘a paradigm shift towards ecosystem management’ in UK coastal and marine governance (interview with author, 2017).

On the one hand, the evolving forms of maritime governance that came with this paradigm shift were taken on willingly by a sequence of UK governments seeking to put the country at the forefront of environmental policy making (Burns et al. 2016). This can be exemplified through the UK’s ambitious adoption of green energy targets, which resulted in the accelerated adoption of wind power, especially offshore, from the mid-2000s onward. From only about 5.5 per cent out of the total in 2008, the UK’s share of renewables rose to 11.3 per cent in 2012 and to 38.6 per cent in 2022 (Department of Energy and Climate Change 2009; Department of Energy and Climate Change 2013; Department for Business, Energy and Industrial Strategy 2022). Offshore wind was a key component of this growth, and although the first UK wind farm was built only in 2000, by 2012 the UK was already the largest global market of offshore wind, representing more than half of Europe’s installed capacity; by 2019, it boasted the world’s largest offshore wind farm, Hornsea 1, replaced in 2022 by its sister project, and Hornsea 2, both located off the coast of Yorkshire (Dawley 2014; Office for National Statistics 2021; Ørsted 2022). Similar levels of ambition were also demonstrated in the context of marine protection with the adoption of a wide-ranging plan to introduce new marine protected areas forming a ‘blue belt’ around the UK (Department for Environment, Food and Rural Affairs 2016).

On the other hand, the implementation of these policies in the UK context was frequently flawed and therefore frequently contested. Blame became discursively shifted onto EU policy by governments unwilling to pay the political cost themselves. At the same time, the EU’s normative link with the ideas behind the policies being implemented—and indeed flaws in the EU’s own policies, especially the CFP—strengthened the conceptual association between the EU, its policies and directives, and the contentiousness of each policy within the paradigm shift. On the coast, perhaps the quintessential example for these dynamics—the role of fishing quota implementation in the UK during the 1990s—has been discussed at length by scholars of marine policy, especially with respect to the confusions over the role of the CFP that prevailed during the Brexit debates (Carpenter et al. 2016; Phillipson and Symes 2018; Stewart and Carpenter 2016; Stewart et al. 2022). The assigning of total allowable catch from individual stocks to countries under the CFP has been shown to get conflated in British popular discourse with the subsequent distribution of this total allowable catch through quotas by the British government itself (Stewart and Carpenter 2016). This distribution of quotas, in the 1990s, particularly affected small-scale fisheries, effectively leaving them out (Carpenter and Kleinjans 2017). As a result, fishermen like James on the Yorkshire shore, who had previously participated in the trawling industry, became essentially pushed out from fishing quota species (interview with author, 2017). This, in turn, meant that smaller-scale fishing communities had less visibility when it came to how national fishing and marine policy was determined.

This reduced visibility, as participants commented in 2017, impacted how local fishing communities reacted to the arrival of offshore wind farms and later MPAs in Yorkshire’s waters. In the Yorkshire context, fisheries advocate Connor illustrated early encounters between developers and the fishing industry had been adversarial as the offshore wind industry had occupied space with little warning and regard for fishing seasons (interview with author, 2017). This had set the tone for local sentiment towards the rapid expansion of new uses of maritime space as part of the paradigm shift in marine governance. Each new use thus had the potential to be seen as a ‘threat of other people turning up and announcing that this bit of the sea is now mine, yes I know you or your family has fished it for 300 years but it belongs to me now and I’m gonna dig it up’ (Connor, interview with author, 2017). This threat, Connor further commented, felt ‘visceral’ to fishing and coastal communities whose local histories, social connections, and livelihoods were all tied to the industry (interview with author, 2017). There was a strengthening sentiment, as researcher Stephen also highlighted that these communities were ‘up against a developer or an organisation that wants to do something in their part of the world, on *their* water, that *they* own, you know—that’s how they felt it’ (interview with author, 2017). For coastal communities, therefore, the fundamental change in maritime spatial governance was perceived as increased enclosure at their livelihoods’ expense. In response, these communities gradually developed the sense that their place in the marine environment has been eroded and that the ongoing policy changes have consistently underprioritised the interests of small-scale fisheries and local communities.

Coastal communities’ sense of place and belonging in their environment, hence, fell under the category of the rapid ‘value shift’ in long-term, often generationally held values that Ford and Goodwin theorised in their thinking of the ‘left behind’ (2014b, 285). In the context of coastal Yorkshire, the strong association with fishing and the industry’s role in generating local community pride made the changing governance values with respect to marine environmental management particularly sensitive. The normative association between the changing governance paradigm and the EU overlapped with the vulnerability of local coastal communities—like all marginalised British communities (Goodwin and Heath 2016)—to narratives recognising ‘left behind’ values. Indeed, in the lead-up to the Referendum, pro-Leave activists found powerful rhetorical ways of evoking these values and the histories behind them. In a ‘Fishing for Leave’ brochure, for example, Ashworth wrote that ‘a great national asset and a whole industry [were] sacrificed as the price of membership. So obsessed was [Prime Minister Edward Heath] with taking the UK into the EEC that he felt this was a price worth paying’ (2016, 4).

The EU remained normatively implicated throughout these dynamics through the UK’s policy implementation. Concerns about the quality of protected areas measures being implemented, for instance, became connected to what was perceived as top-down EU targets for protection. Whereas local policy officer Lauren observed ‘a move towards numbers of sites [in national policy] rather than effective management of sites’, fisheries advocate William claimed that this pressure existed because the UK ‘haven’t implemented enough SPAs as we should have done for Europe’ (interviews with author, 2017). Environmental advocate Peter, meanwhile, critiqued the quality of existing protection and environmental assessments, pointing out that ‘the largest wind farm possibly in the world [is to be built] adjacent to the largest seabird colony in the UK’ (Peter, environmental advocate, 2017). Such concerns compounded on the sentiments of fishermen being pushed out from ‘their’ waters through increased enclosures, as well as on the wider feelings of coastal communities as being increasingly ‘left behind’ economically (given their relative levels of deprivation), politically (in terms of their declining visibility in national politics) and culturally (underrepresented in their connection to the environment).

There are important similarities and continuities between the discursive evocations of fisheries on the Yorkshire coast in the lead-up to both the British accession negotiations to the EEC and in the lead-up to Brexit. Rhetoric from both periods suggests that communities’ sentiments of feeling ‘left behind’ were at least in part inspired by successive UK governments’ flawed implementation of environmental policies as part of a changing paradigm in environmental governance and the associated enclosures of marine space. Second, the EU’s normative association with the shifting marine and environmental governance paradigm provided UK politicians with an opportunity to shift the blame upward. Narratives from both periods demonstrate a willingness among the British political establishment to recognise in fishing and its regional and local history a charged symbol, one expediently capable of resonating with a range of deep-running sentiments among coastal communities. By evoking this symbol, UK politicians capitalised on the shifting and contested nature of environmental identities and beliefs specifically in order to resonate with small communities beset by economic deprivation and cultural isolation, much as theorised by Goodwin and Heath (2016). Thus, narratives in both periods sought to address in particular the invisibility of coastal communities’ environmental meaning and socio-cultural needs in national policy making and implementation against a context of shifting marine governance at the national and supranational level.

## Conclusion

The relevance that environmental meaning has consistently had for coastal communities’ experience of the marine governance transformation on the Yorkshire shore over the decades presents an important contribution to analyses of ‘left behind’ narratives and other socio-cultural explanations for Brexit. The various narratives examined here resonate with the conclusions that scholars like Ford and Goodwin (2014a, b), Goodwin and Heath (2016), and Bolet (2021) have drawn about the significance of social and cultural isolation for voters’ attitudes. Yet these narratives further shine the light on a hitherto overlooked aspect: the environment as a deep-set, fundamental, and contested value adding to these socio-cultural dynamics. As the Yorkshire East Coast demonstrates, the environment can be a key value for the ‘left behind’, one touching on community identity, sense of place and pride, and consequently ways of the local community to relate to national policy.

This direct role of the environment in ‘left behind’ narratives proves especially important to consider in the context of transformations to sustainability. By examining the specific transformation of marine governance post-UNCLOS III on the British East Coast, and taking the transition from sectoral to ecosystem-based marine management to exemplify what an advanced transitional process would look like, this article reaffirms the arguments of scholars like Manuel-Navarrete and Pelling (2015) or Thomas (2021), who have already established that transformations are deeply political processes affected by actors at multiple scales. However, the findings presented here also demonstrate that transformations are political not simply in terms of who gets to participate in or is left out by the resulting regimes of environmental governance but also in terms of *non*-environmental politics. In this instance, the discursive parallels and continuities between the UK’s accession negotiations in the 1970s and Referendum campaigns in 2016 show how the politicisation of environmental values and their mistranslation into environmental governance has had direct and profound effects on the supranational relations between the EU and the UK. This case hence exemplifies how the politics of sustainability transformations can further extend to key non-environmental politics and geopolitical alignments.

These findings have especial implications for advanced environmental states and institutions looking to execute just transitions. They would be directly relevant, for instance, for the EU, which as others have argued often engages with environmental meaning as a source of democratic legitimacy within its member states (Antonova and van Dam 2022; Manners and Murrey 2016) and as a supranational actor on the international stage (Burns 2019; Kelemen and Vogel 2010; Lightfoot and Burchell 2004). With the EU increasingly stating commitments to lead on global sustainable transformations, for example, through its Green Deal (Bloomfield and Steward 2020), the British case presented in this article strongly suggests that successful ‘Green’ or ‘Blue Economy’ agendas must focus on environmental meaning as well as on economics and jobs. In promoting its Green Deal, therefore, the EU will need to ensure that the environmental transformations do not leave various vulnerable socio-ecological communities—including but also going beyond fishing communities—feeling left behind. Evaluation of these policies’ success should consider not only economic indicators but also qualitative assessment of local experiences and shifting environmental values. As the Yorkshire East Coast case shows, failing to do so may have profound implications not only for the intended transformation itself but also in areas outside the environmental politics realm, including European integration.

Looking beyond the specificity of the European or even the maritime context, however, the implications of this article’s findings extend to any context in which transformations in environmental governance are executed. The case examined here illustrates consequences that are not merely political but instead viscerally human. The costs of a transformation’s flawed implementation, as the British case shows, go beyond economics, touching on livelihood, identity, and pride. In this sense, this article joins Bennet et al.’s conclusions (2021) that social justice concerns are as vital as economic considerations to the success of sustainability transformations; but I would further argue for the significance of integrating environmental meaning into transformation policies alongside social justice. This means doing more to elevate shared and individual narratives about environmental values to the policy advisory level. It also means planning environmental transformations along a longer timeframe both before and after policy change has been introduced, allowing for better monitoring of social and environmental justice concerns, as well as for the development of societal resonance with new management priorities. As the accelerating pace of global environmental change prompts ever swifter and more ambitious political solutions, it will be important for social and political science to consider not only how these transformations to sustainability should be executed, but also what it would take for them to be sustained.

## Footnotes

1 All names given for participants in this article are pseudonyms.
